# Draft Genome Sequences of Four Hospital-Associated *Pseudomonas putida* Isolates

**DOI:** 10.1128/genomeA.01039-16

**Published:** 2016-09-29

**Authors:** Mustapha M. Mustapha, Jane W. Marsh, Chinelo D. Ezeonwuka, Anthony W. Pasculle, Marissa P. Pacey, Ashley M. Querry, Carlene A. Muto, Lee H. Harrison

**Affiliations:** a Infectious Diseases Epidemiology Research Unit, University of Pittsburgh, Pittsburgh, Pennsylvania, USA; b Division of Microbiology, University of Pittsburgh Medical Center, Pittsburgh, Pennsylvania, USA; c Division of Hospital Epidemiology and Infection Prevention, University of Pittsburgh Medical Center, Pittsburgh, Pennsylvania, USA

## Abstract

We present here the draft genome sequences of four *Pseudomonas putida* isolates belonging to a single clone suspected for nosocomial transmission between patients and a bronchoscope in a tertiary hospital. The four genome sequences belong to a single lineage but contain differences in their mobile genetic elements.

## GENOME ANNOUNCEMENT

*Pseudomonas putida* is a Gram-negative bacterium belonging to the *fluorescens* group of *Pseudomonas* species and is most commonly found in the soil (1). *P. putida* occasionally causes opportunistic infections of the skin and soft tissue, bacteremia, and may cause serious infections among immunocompromised patients (1–3). A number of hospital-associated cases and small outbreaks of *P. putida* have been described (1, 3–5). *P. putida* is a metabolically versatile pathogen that can withstand a number of physical and chemical stressors, enabling it to survive in a wide range of environmental niches (6–8).

In 2014 to 2015, during the course of routine infection prevention surveillance, four isolates of *P. putida* were identified. One isolate (PSP1) was obtained from a bronchoscope, while the remaining three isolates (PSP2, PSP3, and PSP4) were obtained from clinical cultures from three different patients, raising the possibility of hospital-associated transmission (9). Conventional molecular typing by pulsed-field gel electrophoresis (PFGE) showed 86% band similarity among all four isolates. All isolates had intermediate or full resistance to trimethoprim-sulfamethoxazole, ceftriaxone, and aztreonam.

An isolated colony of each strain was grown overnight on sheep blood agar. Genomic DNA was extracted using the Qiagen DNeasy extraction kit. Illumina Nextera genomic libraries prepared according to the method of Baym et al. (10) were sequenced using the Illumina MiSeq version 2 (500-cycle) kit. The sequencing generated a coverage depth of 43 to 80×. Reads were assembled using SPAdes version 3.5 (11) and annotated using Prokka version 11 (12). Single nucleotide polymorphisms (SNPs) were assessed by mapping PSP2 to PSP4 reads against the PSP1 reference using bwa-mem, GATK tools, and custom python scripts (13, 14). Pairwise SNP distances were calculated from reads using PSP1 as a reference genome.

The four draft genome sequences were assembled into 68 to 155 contiguous nucleotide sequences with 5,332 to 5,396 predicted genes. Core genome phylogenetic analysis showed that all four strains belonged to a distinct phylogenetic cluster that was not closely related to available *P. putida* reference strains; all four genomes were within 61 to 163 pairwise SNP distance. A number of gene differences related to mobile genetic elements were identified. PSP1, PSP3, and PSP4 contained a novel 28-kb prophage-related chromosomal island. Likewise, PSP1, PSP2, and PSP3 contained a putative 50-kb plasmid that had less than 50% similarity with any known plasmid sequence.

These findings are in keeping with the transmission and microevolution of a unique clone of *P. putida* containing previously uncharacterized mobile genetic elements within the hospital. Continued surveillance and monitoring of patients and the hospital environment are required to monitor the evolution of *P. putida* and other strains associated with hospital transmission. Whole-genome sequence (WGS) analysis is rapidly becoming the gold standard for hospital infection surveillance and for the investigation of suspected health care-associated transmission of infectious pathogens (15). The availability of comparison strains, such as those presented in this work, is essential for comparative genomic analyses of nosocomial pathogens.

### Accession number(s).

This whole-genome shotgun project has been deposited at GenBank under the accession numbers MCBG00000000, MCBH00000000, MCBI00000000, and MCBJ00000000. The version described here is the first version, with MCBG01000000, MCBH01000000, MCBI01000000, and MCBJ01000000, respectively.

## References

[1] Yoshino Y, Kitazawa T, Kamimura M, Tatsuno K, Ota Y, Yotsuyanagi H 2011 *Pseudomonas putida* bacteremia in adult patients: five case reports and a review of the literature. J Infect Chemother 17:278–282. doi:10.1007/s10156-010-0114-0. 20809240

[2] Bogaerts P, Huang TD, Rodriguez-Villalobos H, Bauraing C, Deplano A, Struelens MJ, Glupczynski Y 2008 Nosocomial infections caused by multidrug-resistant *Pseudomonas putida* isolates producing VIM-2 and VIM-4 metallo-beta-lactamases. J Antimicrob Chemother 61:749–751. doi:10.1093/jac/dkm529. 18238886

[3] Thomas BS, Okamoto K, Bankowski MJ, Seto TB 2013 A lethal case of bacteremia due to soft tissue infection. Infect Dis Clin Pract 21:147–213. 10.1097/IPC.0b013e318276956b PMC3673730 23750097

[4] Hardjo Lugito NP, Nawangsih C, Moksidy JC, Kurniawan A, Tjiang MM 2015 Diabetic foot gangrene patient with multi-drug resistant *Pseudomonas putida* infection in Karawaci District, Indonesia. J Glob Infect Dis 7:37–39. doi:10.4103/0974-777X.146378. 25722620 PMC4338450

[5] Kim SE, Park SH, Park HB, Park KH, Kim SH, Jung SI, Shin JH, Jang HC, Kang SJ 2012 Nosocomial *Pseudomonas putida* bacteremia: high rates of carbapenem resistance and mortality. Chonnam Med J 48:91–95. doi:10.4068/cmj.2012.48.2.91. 22977749 PMC3434797

[6] Nelson KE, Weinel C, Paulsen IT, Dodson RJ, Hilbert H, Martins dos Santos VA, Fouts DE, Gill SR, Pop M, Holmes M, Brinkac L, Beanan M, DeBoy RT, Daugherty S, Kolonay J, Madupu R, Nelson W, White O, Peterson J, Khouri H, Hance I, Chris LP, Holtzapple E, Scanlan D, Tran K, Moazzez A, Utterback T, Rizzo M, Lee K, Kosack D, Moestl D, Wedler H, Lauber J, Stjepandic D, Hoheisel J, Straetz M, Heim S, Kiewitz C, Eisen JA, Timmis KN, Dusterhoft A, Tummler B, Fraser CM 2002 Complete genome sequence and comparative analysis of the metabolically versatile *Pseudomonas putida* KT2440. Environ Microbiol 4:799–808. doi:10.1046/j.1462-2920.2002.00366.x. 12534463

[7] Wackett LP 2003 *Pseudomonas putida*—a versatile biocatalyst. Nat Biotechnol 21:136–138. doi:10.1038/nbt0203-136. 12560839

[8] Nikel PI, Martínez-García E, de Lorenzo V 2014 Biotechnological domestication of pseudomonads using synthetic biology. Nat Rev Microbiol 12:368–379. doi:10.1038/nrmicro3253. 24736795

[9] Querry A, Metzger A, Galdys A, Pasculle W, Delgado E, Donahoe M, Muto CA. *Pseudomonas putida* cluster associated with a contaminated bronchoscope in a medical ICU at the University of Pittsburgh Medical Center, Open Forum Infectious Diseases, IDWeek 2016 Abstracts, in press.

[10] Baym M, Kryazhimskiy S, Lieberman TD, Chung H, Desai MM, Kishony R 2015 Inexpensive multiplexed library preparation for megabase-sized genomes. PLoS One 10:e0128036. doi:10.1371/journal.pone.0128036. 26000737 PMC4441430

[11] Bankevich A, Nurk S, Antipov D, Gurevich AA, Dvorkin M, Kulikov AS, Lesin VM, Nikolenko SI, Pham S, Prjibelski AD, Pyshkin AV, Sirotkin AV, Vyahhi N, Tesler G, Alekseyev MA, Pevzner PA 2012 SPAdes: a new genome assembly algorithm and its applications to single-cell sequencing. J Comput Biol 19:455–477. doi:10.1089/cmb.2012.0021. 22506599 PMC3342519

[12] Seemann T 2014 Prokka: rapid prokaryotic genome annotation. Bioinformatics 30:2068–2069. doi:10.1093/bioinformatics/btu153. 24642063

[13] DePristo MA, Banks E, Poplin R, Garimella KV, Maguire JR, Hartl C, Philippakis AA, del Angel G, Rivas MA, Hanna M, McKenna A, Fennell TJ, Kernytsky AM, Sivachenko AY, Cibulskis K, Gabriel SB, Altshuler D, Daly MJ 2011 A framework for variation discovery and genotyping using next-generation DNA sequencing data. Nat Genet 43:491–498. doi:10.1038/ng.806. 21478889 PMC3083463

[14] Li H 2013 Aligning sequence reads, clone sequences and assembly contigs with BWA-MEM. arXiv arXiv:1303.3997.

[15] Arnold C 2015 Outbreak breakthrough: using whole-genome sequencing to control hospital infection. Environ Health Perspect 123:A281–A286. 26523889 10.1289/ehp.123-A281 PMC4629729

